# Lymphangioleiomyomatosis and Pregnancy—Do We Have All the Answers for a Woman Who Desires to Conceive?—Literature Review

**DOI:** 10.3390/cancers17020323

**Published:** 2025-01-20

**Authors:** Ancuta-Alina Constantin, Andreea Dumitrita Gaburici, Andreea Nicoleta Malaescu, Ana-Luiza Iorga, Christiana Diana Maria Dragosloveanu, Mircea-Octavian Poenaru, Gabriel-Petre Gorecki, Mihaela Amza, Mihai-Teodor Georgescu, Ramona-Elena Dragomir, Mihai Popescu, Romina-Marina Sima

**Affiliations:** 1 Department of Cardio-Thoracic Pathology, “Carol Davila” University of Medicine and Pharmacy, 050474 Bucharest, Romania; ancuta-alina.constantin@umfcd.ro (A.-A.C.); andreea-dumitrita.slabu@rez.umfcd.ro (A.D.G.); andreea-nicoleta.malaescu@rez.umfcd.ro (A.N.M.); ana-luiza.iorga@drd.umfcd.ro (A.-L.I.); 2 Institute of Pneumology “Marius Nasta”, 050159 Bucharest, Romania; 3 Department of Ophthalmology, “Carol Davila” University of Medicine and Pharmacy, 050474 Bucharest, Romania; christiana.dragosloveanu@umfcd.ro (C.D.M.D.); mihai.georgescu@umfcd.ro (M.-T.G.); ramona-elena.dragomir@drd.umfcd.ro (R.-E.D.); 4 Department of Ophthalmology, Clinical Hospital for Ophthalmological Emergencies, 010464 Bucharest, Romania; 5 Department of Obstetrics and Gynecology, “Carol Davila” University of Medicine and Pharmacy, 020021 Bucharest, Romania; mihalea.amza@umfcd.ro (M.A.); romina.sima@umfcd.ro (R.-M.S.); 6 “Bucur” Maternity, Saint John Hospital, 012361 Bucharest, Romania; 7 Department of Anesthesia and Intensive Care, Faculty of Medicine, “Titu Maiorescu” University, 031593 Bucharest, Romania; gabriel.gorecki@prof.utm.ro; 8 Department of Anesthesia and Intensive Care, CF2 Clinical Hospital, 011464 Bucharest, Romania; 9 “Prof. Dr. Al. Trestioreanu” Oncology Discipline, “Carol Davila” University of Medicine and Pharmacy, 020021 Bucharest, Romania; 10 Department of Anesthesia and Intensive Care, “Carol Davila” University of Medicine and Pharmacy, 020021 Bucharest, Romania; mihai.popescu@umfcd.ro; 11 Bucharest University Emergency Hospital, 169 Splaiul Independentei, 050098 Bucharest, Romania

**Keywords:** lymphangioleiomyomatosis, pregnancy, complications, sirolimus

## Abstract

Lymphangioleiomyomatosis (LAM) is a rare, progressive, and poor-prognosis systemic disorder that primarily affects women of reproductive age, with a higher prevalence among individuals of Caucasian origin. However, there are limited reliable data on the prevalence of LAM during pregnancy. The fulminant respiratory clinical presentation that often includes progressive dyspnea on exertion, cough, or hemoptysis, frequently complicated by pneumothorax, and the increased risk of spontaneous abortion due to increased estrogen and progesterone production during gestation, are arguments that most often make the diagnosed woman avoid pregnancy. Elevated levels of vascular endothelial growth factor D (VEGF-D), decline in respiratory function, and radiological findings are sufficient arguments in favor of the diagnosis in the pregnant woman. Sirolimus, an mTOR inhibitor, has demonstrated effectiveness in slowing the decline of lung function. Although sirolimus treatment is often recommended to be discontinued before conception due to the increased risk of fetal growth restriction, maintaining a dose level of <5 pcg/mL, with serum drug levels of 3–5 pcg/L, has been considered safe. Given the potential risks, individualized decisions about pregnancy are advised for patients with LAM. For those who choose to proceed, close monitoring by a multidisciplinary team is essential to manage complications effectively. Ongoing research aims to provide clearer guidance to optimize outcomes for both mother and child.

## Simple Summary

In recent years, we have witnessed an increase in the prevalence of LAM in the female population as a result of the increased availability of diagnostic methods on a large scale definitive diagnosis of LAM requires pulmonary biopsy, which reveals characteristic cellular morphology, including smooth muscle fiber proliferation. In cases where high-resolution computed tomography (HRCT) findings are highly suggestive, elevated levels of vascular endothelial growth factor D (VEGF-D), secreted by LAM cells within the lung, may serve as a non-invasive alternative to biopsy in pregnancy. Although Sirolimus has proven to be an appropriate treatment that could be safely administered in certain amounts during pregnancy, the high number of reported adverse effects on the fetus and the emergence of Everolimus, a drug with potential benefits, are arguments in favor of the need for more studies to establish new treatment options.

## 1. Introduction

LAM is a rare and progressive pulmonary disorder that predominantly affects women of childbearing age. This condition is marked by an abnormal growth of smooth muscle cells that invade lung tissues, including the airways and blood and lymph vessels, causing lung destruction and resulting in airflow and vessel obstruction, which generally progresses to respiratory failure. Beyond the lungs, LAM may also involve the kidneys and lymphatic system, contributing to its multisystemic impact.

LAM may manifest either as a sporadic condition (S-LAM) or in conjunction with tuberous sclerosis complex (TSC), known as TSC-LAM. Although it frequently appears in tuberous sclerosis—a condition more often associated with a mutation in the TSC2 tumor suppressor gene rather than TSC1, which aberrantly activates the mTOR pathway—LAM is commonly diagnosed in its sporadic form (S-LAM), primarily affecting the lungs [1]. Histopathologically, LAM is considered an estrogen-dependent mesenchymal tumor composed of spindle-shaped and epithelioid cells expressing specific markers on their surface typical of smooth muscle cells [2].

Despite numerous debates on whether lymphangioleiomyomatosis can be classified as a neoplasm, its pattern of lymphatic spread, multisystem involvement, evidence of LAM cells in blood and urine, and reports of recurrence of cystic lesions in transplanted lungs support this classification [3,4,5]. Since LAM muscle cells express two lymphangiogenic growth factors—vascular endothelial growth factor C (VEGF-C) and vascular endothelial growth factor D (VEGF-D)—and disseminate through the lymphatic system, LAM is considered a low-grade metastatic neoplasm.

A characteristic feature of lymphangioleiomyomatosis is the presence of cystic lesions containing air or fluid (serocitrin/lymphatic), which result in significant clinical symptoms, such as reduced exercise tolerance. These changes can be objectively measured through paraclinical findings as a decrease in forced expiratory volume in one second (FEV1) and diffusing capacity of the lungs for carbon monoxide (DLCO) [6,7]. Potential complications that may arise during pregnancy, likely exacerbated by hormonal changes, include pneumothorax, spontaneous abortion, and premature birth. These complications present a challenge for clinicians in assessing whether a patient can hope for a pregnancy that concludes without major complications under appropriate treatment [8]. Diagnosing LAM during pregnancy is a dramatic event due to the negative prognosis for both the mother and fetus.

## 2. Epidemiological Data

The complexity of establishing a diagnosis, the need for imaging investigations, the clinician’s challenge in distinguishing lymphangioleiomyomatosis lesions from other pathologies, as well as the lack of national registries in developing and underdeveloped countries, are all negative prognostic factors that hinder the availability of precise data. Although over 80% of women diagnosed with TSC also have LAM, and S-LAM is the most common form, it should be noted that men with TSC may also develop LAM [1]. In recent years, there has been an observed increase in LAM prevalence, likely due to improved patient access to imaging methods; however, substantial variability persists in available epidemiological data. In a meta-analysis by Suter et al. that reviewed 234 articles on S-LAM prevalence, excluding TSC-LAM cases, it was estimated that 3.03 women per million are affected by this disease [9]. These findings align with those of Harknett et al., who estimated 3.4–7.8 cases per million based on a large sample of 1001 cases from three national registries [10]. More recent studies, however, have raised questions about LAM’s rarity. A study by Lynn, covering four Nordic countries, significantly increased this estimate to 23.5 cases per million women. While there is no evidence that LAM prevalence varies by region or race, the findings of Lynn et al. align with those from a Japanese national registry study, which estimated a prevalence of 28.7 cases per million women from 2013 to 2018 [11,12]. The impact on women of reproductive age and the potential adverse effects of pregnancy underscore the need to update epidemiological data, which is essential for increasing physician awareness and guiding primary healthcare policies. These policies should prioritize comprehensive screening strategies for women of reproductive age who have a family or personal history of progressive dyspnea, spontaneous pneumothorax, and angiolipomas that include imaging (chest tomography), serology (VEGF-D testing), and concomitant development of new treatments to improve both life expectancy and quality of life [13]. Although we lack precise data on LAM epidemiology in pregnancy, it is estimated that over a third of diagnosed women have never been pregnant—either due to specialist advice (25%) or concerns about disease progression (30%), compared with 15% in the general population. Most surveyed women with LAM were diagnosed after pregnancy (86.82%), 7.3% during pregnancy, and 5.83% became pregnant after their diagnosis [14]. In a study by Shen et al., which applied a pregnancy-related questionnaire to 94 patients with LAM, only 30 had been pregnant, with a 30% term birth rate [8].

## 3. Pathogenic Pathways

Studies have shown the expression of estrogen receptors (ER) and progesterone receptors (PR) in LAM cells, indicating that hormonal changes during pregnancy can directly affect disease dynamics [15]. This rare and progressive disease arises from the estrogenic effect on cells that have undergone aberrant somatic changes due to mutations in the TSC1 (hamartin) or TSC2 (tuberin) tumor suppressor genes. These mutations inappropriately stimulate the mTOR (mechanistic/mammalian target of rapamycin complex 1) pathway. Understanding LAM pathogenesis has required decades of research into the mTOR pathway and its physiological role. While mTOR is expressed intracellularly in two complexes, mTORC1 and mTORC2, it has been shown that mTORC1 is specifically implicated in LAM pathogenesis. The mTORC1 complex, considered crucial in LAM, regulates cell growth and is stimulated by estrogen and other growth factors in the presence of nutrients (amino acids, glucose, lipids). Estrogenic stimulation activates the mTORC1 pathway through phosphatidylinositol-3 kinase (PI3K) by binding phosphatidylinositol-dependent kinase-1 (PDK1) with Akt, which suppresses TSC1 and TSC2 [16]. Proteins Rags and Rheb interact with mTORC1 on the lysosomal surface, facilitating its translocation intralysosomally in the presence of increased amino acids [17,18]. Mutations in the TSC1 and TSC2 genes prevent proper downregulation, as the necessary protein for converting Rheb and Rag from the active GTP-bound form to the inactive GDP-bound form is absent [19]. Glutamine/leucine transporters acting via antiport systems—where glutamine efflux and leucine influx occur—further stimulate mTORC1 by increasing intralysosomal leucine levels [20]. While TSC1 and TSC2 mutations are considered the primary mechanisms in LAM, multiple studies have suggested that overexpression of these antiport systems may have a neoplastic role [19]. Elevated leucine levels promote the phosphorylation of several ribosomal proteins, including 4EBP1 (eukaryotic translation initiation factor 4E binding protein 1) and S6K1 (S6 kinase beta-1), both implicated in LAM’s mechanisms [21,22]. Ribosomal proteins 4EBP1 and S6K1 are essential for survival, supporting cell proliferation, size increase, protein synthesis (S6K1), angiogenesis, and cell survival (4EBP1) [23].

Increased estrogen levels during pregnancy, along with the mTOR pathway’s essential role in placental function and development, explain the exacerbation of LAM in pregnant women [24]. Although the mechanism above explains LAM cell formation, the parenchymal remodeling characterized by numerous cysts results from a synergistic action between the mTOR and WNT pathways, adding complexity to the disease and complicating the development of future therapies. The WNT pathway plays a role in pulmonary parenchymal homeostasis, stimulating cell proliferation and differentiation mainly during organogenesis and less so in adulthood [25]. Experimental work by Obraztsova et al. demonstrated that excessive activation of the WNT-mTOR pathway leads to defective cystic remodeling due to impaired mesenchymal-epithelial transition. This effect is attributed to the prolonged proliferative capacity of type II alveolar cells and an increased number of type 1/type 2 transitional cells, which is exacerbated during pregnancy [26]. The progression of LAM lesions during sirolimus treatment, which inhibits the mTOR pathway, may be partially explained by continuous WNT pathway activation despite treatment [27]. Histopathologically, LAM cystic lesions consist of cellular clusters of immature smooth muscle cells with a perivascular phenotype, expressing smooth muscle α-actin, phosphoribosomal protein S6, estrogen receptor *alpha*, and progesterone receptor on their surfaces [2,28]. Initially, LAM cells form nodules composed of fibroblasts and lymphatic endothelial cells recruited through VEGF-D stimulation, gradually enlarging as elastin and collagen fibers degrade and type II alveolar cells proliferate [2]. The heterogeneity of LAM arises from the diversity of affected organs, with specific patient-dependent characteristics explained by the fact that, while TSC-LAM shows homogeneous TSC deficiency, the sporadic form is characterized by cellular mosaicism [29].

**Progesterone**, while less extensively studied, also appears to influence LAM progression during pregnancy. Studies have demonstrated that PR expression is often higher than ER in LAM cells, particularly in early-stage disease [15]. However, progesterone’s role remains controversial, as progestins have demonstrated limited therapeutic efficacy and potential proliferative effects through the activation of pro-growth pathways like MAPK (Mitogen-Activated Protein Kinase) and PI3K/Akt/mTOR (Phosphoinositide 3-Kinase/Protein Kinase B/Mechanistic Target of Rapamycin) [15].

The combination of elevated **estrogen** and **progesterone** during pregnancy creates a hormonal environment conducive to LAM cell proliferation and metastasis. Current treatments, such as estrogen receptor antagonists and mTOR inhibitors, have not yet proven consistently effective in mitigating pregnancy-related complications in LAM, underscoring the need for targeted therapies addressing the unique hormonal milieu of pregnancy [30].

## 4. Clinical Picture

Although LAM has a relatively strict definition in terms of pathogenic mechanisms, the clinical spectrum that may raise suspicion of the disease is broad [31]. In this context, the diagnostic pathway can be subtle, often beginning with an asymptomatic patient—a scenario reported in up to 18% of cases [32]—where pulmonary cysts are incidentally identified on imaging [33]. More rarely, the onset of the disease can be dramatic, presenting with either pronounced symptoms or life-threatening complications [31].

Since LAM predominantly affects women of childbearing age, pregnancy becomes a topic of particular interest, requiring a personalized decision made jointly by the patient and her attending physician. However, pregnancy should be discouraged in cases of severe disease involvement [8]. While patients with LAM may have consecutive pregnancies without significant complications, it is generally accepted that pregnancy amplifies symptoms and increases the risk of complications [34]. Additionally, a study of adult women diagnosed with LAM in the United States of America found that respiratory symptoms occurring during pregnancy led to a LAM diagnosis in 34% of cases [35].

Most commonly, patients with LAM present at disease onset with exertional dyspnea, a symptom reported in 60% of cases [33], but which may occur in up to 88.2% of patients with diffuse cystic lung involvement [35]. This can initially lead to misdiagnosis, delaying appropriate diagnostic and therapeutic management [31]. Other frequently identified symptoms include cough—present in approximately one-quarter of diagnosed patients, either dry or productive—and fatigue, occurring in about 18% of cases. Isolated cases have reported episodes of hemoptysis, wheezing, and chest pain [31]. During pregnancy, respiratory symptoms often intensify, with dyspnea being particularly prominent [14].

A significant factor impacting quality of life is pleural involvement, which includes the most common complications during pregnancy [8]. This can manifest as pneumothorax, which occurs in over one-third of the general population with LAM (36%) [33], with an annual incidence of 8% [34], compared to a significantly higher frequency during the gestational period (29%). The peak incidence of this condition occurs during the second trimester of pregnancy (59%) [36]. During labor, there is a considerable risk of pneumothorax, which, if it occurs, necessitates the temporary insertion of a drainage tube until definitive surgical treatment can be performed [35]. Isolated cases, secondary to these complications or the required treatment, have reported a high incidence of spontaneous abortion, emergency cesarean section, or induced termination of pregnancy [35]. In patients with severe lung involvement due to large cysts or significant impairment of lung function, the risk of pneumothorax is significantly increased during air transport [37,38]. Therefore, it is advisable that, at least during pregnancy, women with LAM be counseled against air travel [39]. Among the spectrum of pleural involvement, chylothorax is also noteworthy, occurring less frequently, with a prevalence of 3% in the general population with LAM [33].

Extrapulmonary involvement can manifest as angiomyolipomas in approximately half of the patients (45%), or, occasionally, as lymphangioleiomyomas (18%) [33]. These conditions can lead to a complex clinical picture, often presenting with nonspecific abdominal pain, hematuria, chronic kidney disease, or lymphatic edema of the lower limbs [32]. Most angiomyolipomas are found in the kidneys; however, they can also occur in the uterus [40], liver, or spleen [41]. Among pregnant women known to have LAM, there have been reports of either newly diagnosed angiomyolipomas during pregnancy or significant progressive growth of previously known angiomyolipomas [41,42,43,44,45]. In such cases, the risk of angiomyolipoma rupture increases, manifesting as significant abdominal pain, hematuria, hypotension, and shock. This risk is correlated with accelerated maternal blood circulation and hormonal changes that occur during pregnancy [43], as well as the increased volume of the uterus, exerting pressure on the affected areas [44].

An important consideration for patients with LAM is the reported disturbance in sleep quality, which is closely linked to the degree of pulmonary functional impairment. A study conducted on 15 individuals diagnosed with sporadic LAM showed that up to half of the patients reported poor sleep quality or unrefreshing sleep at least two nights a week, obstructive sleep apnea during REM sleep in more than 40% of cases, and nocturnal hypoxemia without meeting the criteria for respiratory failure in a quarter of the patients. Additionally, depression and anxiety are noted as secondary symptoms associated with compromised quality of life [46]. Among women with obstructive sleep apnea, there is an increased risk of specific pregnancy complications, such as gestational diabetes (19%), pregnancy-induced hypertension (9%), and preeclampsia (14.1%). Maternal complications have also been identified, although less frequently, including pulmonary edema, congestive heart failure, or cardiomyopathy, as well as fetal complications such as intrauterine growth restriction or late fetal death [47].

## 5. Paraclinical Investigations

Imaging investigations such as plain chest X-rays or computed tomography (CT) scans should be avoided during pregnancy due to their significant effects on fetal development, especially in the first trimester. In this context, the consequences can be significantly detrimental, potentially leading to fetal malformations affecting the eyes, skeleton, or genital tract, as well as developmental anomalies or neurological effects such as microcephaly or intellectual disability. However, X-rays may be necessary during pregnancy in cases of persistent cough lasting more than 8 weeks or for patients reporting dyspnea, hemoptysis, or chest pain [48].

## 6. Chest X-Ray

Chest X-ray can provide useful information with signaling value, though a normal chest X-ray does not rule out LAM. Radiographic changes may be subtle or nonspecific, particularly in the early stages. Identifiable features include accentuated pulmonary interstitium, reticulo-nodular opacities, lymphadenopathy, and hyperinflation [49]. Pulmonary hyperinflation is noted in approximately half of the patients. Interstitial reticular opacities are seen in about 66% of cases, primarily affecting the lower regions of the lung fields, where cystic formations can also be identified radiologically. Additionally, other alterations frequently detected on plain chest X-rays include pneumothorax and pleural effusion, each occurring in roughly half of the cases [50].

## 7. Thoracic Computed Tomography

The imaging investigation using thoracic computed tomography (CT) is considered the foundation for suspecting a diagnosis of LAM. This examination is performed either to screen women of childbearing age with tuberous sclerosis to determine the etiology of respiratory symptoms arising secondary to the specific cystic structural changes associated with the disease or to assess complications such as pneumothorax or chylothorax [49]. During pregnancy, it is believed that the radiation dose associated with thoracic examinations is significantly lower than that of abdominal examinations, as the fetus is not directly exposed [48].

The cysts identified in LAM, which can be detected in up to 100% of cases in some studies [50], have thin, uniform walls and vary in size from 2 mm to several centimeters [51]. When there are at least 10 cysts, they fulfill part of the diagnostic criteria for LAM [31]. These cysts are thought to develop due to air trapping caused by the proliferation of peribronchial smooth muscle cells [52]. They can be diffusely distributed throughout all areas of the pulmonary parenchyma, with the middle third of the lung primarily involved, and may appear round, oval, or polygonal in shape [50].

In this context, the differential diagnosis includes conditions that may mimic the lesions identified on imaging, such as emphysema, Langerhans cell histiocytosis, amyloidosis, lymphoid interstitial pneumonia, and Birt–Hogg–Dubé syndrome [49].

Small-sized centrilobular pulmonary nodules may be attributed to hyperplasia of muscle cells or pneumocytes [52]. This feature can be identified in up to approximately one-third of patients, with its prevalence varying by race [53]. Less commonly, ‘ground-glass’ opacities may be identified, which correspond to the proliferation of muscle cells or alveolar hemorrhage [52].

Pneumothorax can be detected on imaging in about 50% of patients and may be unilateral or bilateral. Lymphatic obstruction can lead to the development of chylothorax or thickening of the pulmonary septa. Involvement of the pulmonary vessels may be indicated by the thickening of the arterial walls or venous occlusion [52]. Among the lymphadenopathic findings, those with mediastinal localization have been detected most frequently [53].

Lesions identified through imaging may evolve in number and size during pregnancy, secondary to the cessation of specific treatment or hormonal variations; however, this phenomenon has been shown to be potentially reversible [54].

## 8. Thoracic Ultrasonography

In cases of suspected pulmonary conditions, chest ultrasound can be utilized as a first-line method for pregnant women, as it is considered a non-invasive technique for the fetus. However, there is a reported risk of thermal injury to the exposed maternal tissues and the fetus [48]. While pulmonary cysts cannot be visualized using ultrasound, the technique remains valuable for diagnosing complications that are predominantly pleural in nature, such as pleural effusions, pleural thickening, and pulmonary consolidations [55,56].

## 9. Abdominopelvic Imaging Investigations

Although abdominopelvic CT can identify angiomyolipomas, lymphangioleiomyomas, and lymphadenopathies [31], it should be avoided during pregnancy due to the direct exposure of the fetus [48]. It is significantly superior to ultrasound in terms of specificity and sensitivity, as it can detect tumors smaller than 1 cm [31]. Studies have shown that renal angiomyolipomas can be detected in nearly half of known patients with LAM, while abdominal lymphadenopathies, whether single or multiple, occur in approximately 40% of cases. Lymphangioleiomyomas are identified in about 16% of cases. Additionally, ascitic fluid and dilation of the thoracic duct can also be observed, each occurring with a frequency of 9% [57].

## 10. Respiratory Functional Tests

One of the tests used to evaluate respiratory function in patients with LAM is spirometry, which may show normal values in up to one-third of cases, especially in patients with LAM associated with tuberous sclerosis [58]. Typically, a reduced Tiffneau index is observed, suggesting obstructive ventilatory dysfunction. Bronchial hyperreactivity may also be present, evidenced by partial reversibility after the administration of a bronchodilator, leading to an increase in the forced expiratory volume in one second (FEV1) [59] in approximately half of the cases [60].

Plethysmography detects a residual volume exceeding 120% of the predicted value due to air trapping. The diffusing capacity of carbon monoxide across the alveolar-capillary membrane (DLCO) is frequently altered before the impairment of FEV1 [58]. FEV1 decreases by approximately 118 mL per year in patients with LAM [61], compared to values recorded in healthy individuals (20–30 mL per year) [59]. Studies have shown an accelerated decline in both FEV1 and DLCO during pregnancy, with FEV1 decreasing by an average of 15% and DLCO by 9%. Therefore, although women with LAM can carry a pregnancy to term, the decline in pulmonary function should not be overlooked, even if values prior to planning a pregnancy may appear normal [62]. Arterial blood gas analysis can be performed at the time of diagnosis to establish a baseline regarding the degree of pulmonary impairment, indicating either normal values or the presence of hypoxemia, with or without hypercapnia. This analysis should only be repeated when transcutaneous oxygen levels fall below 95% in ambient air. The six-minute walk test serves a similar purpose, as desaturation that occurs during exertion may correlate with the severity of the disease [59].

## 11. Biological Tests

A serum vascular endothelial growth factor (VEGF-D) level greater than 800 pg/mL exhibits high sensitivity and specificity for diagnosing LAM [49] and correlates with the severity of pulmonary functional impairment (Tiffneau index, residual volume, total lung capacity, DLCO) [51]. VEGF-D levels are significantly elevated in patients with LAM compared to individuals with other lung diseases or healthy subjects. A study involving a Japanese population reported a mean serum VEGF-D level of 1568 pg/mL in cases of sporadic LAM and 4485 pg/mL in LAM associated with tuberous sclerosis, compared to significantly lower values in other pulmonary conditions (399 pg/mL) and in healthy individuals (392 pg/mL). Thus, VEGF-D levels can be used as a marker in LAM, with values progressively decreasing after the initiation of sirolimus therapy. These levels remain relatively constant during pregnancy but may show a reversible increase, particularly at the time of delivery [63].

In addition to VEGF-D, further investigations are necessary to exclude α1-antitrypsin deficiency, which predisposes individuals to emphysema (via enzymatic measurement), connective tissue diseases (via rheumatoid factor, antinuclear antibodies, anti-Ro, and anti-La antibody assays), and lymphoproliferative disorders such as multiple myeloma (via serum and urinary electrophoresis) [48,64].

Loss of heterozygosity in one of the TSC genes, particularly TSC2 (TSC2-LOH), has been identified in LAM cells present in blood, urine, or chylous fluid, suggesting its potential utility as a biomarker in clinical practice. However, further research is required, especially given the current lack of specificity and correlation with disease severity or extrapulmonary manifestations [31]. When clinical suspicion of TSC arises, genetic testing for mutations in the TSC1 and TSC2 genes is recommended. In cases of sporadic LAM, genetic testing is not typically advised, although somatic mutations in these genes may be present exclusively in LAM cells [59].

## 12. Histopathological Examination

During pregnancy, interventions necessary for a histopathological diagnosis are recommended to be postponed until after the pregnancy has reached term, particularly since they are not part of the criteria required for diagnosing the disease. However, it is important to emphasize that the preferred method for obtaining a histopathological sample is pulmonary biopsy via video-assisted thoracoscopic surgery (VATS). Equally valid alternatives include the analysis of lymph node fragments or an excised piece of angiomyolipoma [59]. Endobronchial ultrasound-guided transbronchial biopsy is also an accepted option, although it is less commonly associated with significant complications, such as pneumothorax or hemorrhage. Limited bleeding is frequently reported and can usually be managed easily with a local injection of epinephrine [65]. Cytological analysis of pleural fluid or ascitic fluid can enhance the diagnostic process by identifying LAM cells in 15% of patients [59].

## 13. Specific Challenges for Pregnancy—Treatment and Outcome

Before discussing the therapeutic implications of pregnancy in LAM, several noteworthy aspects should be considered. Therapeutic management during pregnancy begins with family planning, which includes providing information to the patient and her family regarding the potential for disease progression or the onset of complications during pregnancy, as well as the genetic implications this condition may have for the future child. Multidisciplinary clinical, imaging, and functional investigations should be conducted prior to pregnancy to document the progression of the disease. Furthermore, the use of mTOR inhibitors should either be discontinued during pregnancy or administered intermittently [59].

Until recently, treatment for LAM was primarily supportive, focusing on bronchodilator therapy, supplemental oxygen, complication management, and lung transplantation [66]. However, in-depth studies of mutations in the TSC2 gene, which trigger activation of the mammalian target of rapamycin (mTOR), have enhanced the management of these patients. In recent years, treatments that focus on inhibitors of this pathway have led to significantly better outcomes regarding disease progression [67]. Given that the therapy aims to enhance the quality of life by improving pulmonary function, one of the eligibility criteria for initiating treatment with sirolimus is based on functional pulmonary impairment, which includes an FEV1 below 70% of the predicted value. Another criterion is the presence of chylous fluid accumulation, for which pharmacological treatment is preferred over invasive interventions [68].

The MILES study is one of the most well-known trials conducted to establish the therapeutic efficacy of mTOR inhibitors. This randomized, double-blind trial included 89 patients with pulmonary function impairment related to LAM (FEV1 < 70% of the predicted value) and monitored them over a one-year period. However, one of the exclusion criteria was the presence of an active pregnancy. The results demonstrated that patients treated with sirolimus experienced stabilization of pulmonary function during therapy, with relatively constant FEV1 values and an increase in FVC compared to placebo. These findings underscore the importance of therapy in moderate to severe forms of the disease. The most common adverse reactions included diarrhea, nausea, hypercholesterolemia, acneiform rash, and edema of the lower extremities. Although less common, pericarditis and atrial arrhythmias have a significantly negative impact on patient health [69]. Additionally, it has been shown that sirolimus reduces the incidence of pneumothorax, with the risk of recurrence at 5 years being 80% lower in individuals receiving treatment [70].

Studies have been conducted during pregnancy to assess the effects of stopping sirolimus treatment versus continuing it. It is generally recommended to discontinue treatment 12 weeks prior to pregnancy, throughout pregnancy, and during breastfeeding. An increased risk of miscarriage or preterm birth has been reported in patients with pregnancy who continued the medication, although no malformations or adverse effects on the fetus were identified [8]. While the teratogenic risk has not been established, sirolimus may potentially cause intrauterine growth restriction due to placental epigenetic modulation and inhibition of the mTOR pathway [71]. Nevertheless, several case reports have highlighted the absence of adverse effects on both the mother and the fetus [72,73,74]. Therefore, it is considered acceptable to use sirolimus during pregnancy when the benefits outweigh the potential risks [8], at a lower dose of <5 pcg/mL, with serum drug levels of 3–5 pcg/L [72]. Since Zhou et al. [39], in a mini-review, evaluated pregnancies occurring in the context of a pre-existing LAM diagnosis, with patients receiving sirolimus starting from the time of diagnosis, we have identified 10 case reports for comparison where the diagnosis was established during pregnancy (Table 1). Of these 10 patients, 9 did not receive sirolimus before or during pregnancy, while the 10th patient was administered sirolimus intermittently during pregnancy. The success rate for healthy newborns was 100% in the mini-review, compared to 90% in the cases from Table 1. Additionally, the prevalence of pregnancy complications was lower in the mini-review, with 3 (27%) cases of pneumothorax compared to 6 (60%) cases of pneumothorax in the Table 1 cases.

Regarding everolimus, another mTOR inhibitor, insufficient studies exist to establish its effects on pregnancy; however, paradoxically, it has been shown to have the potential to increase HLA-G, a protein that helps maintain maternal–fetal immune tolerance [84].

An alternative therapeutic option that has been considered is doxycycline, a tetracycline that inhibits matrix metalloproteinases (MMPs) and proteolytic enzymes involved in cyst formation, although the exact mechanism is not fully understood. However, it is not recommended for the treatment of patients with LAM, as no significant therapeutic benefit has been demonstrated [85]. Furthermore, it should be avoided in pregnant women due to the risk of adverse effects, including preterm birth, spontaneous abortion, intrauterine death, or congenital anomalies [86].

Metformin, a therapeutic agent from the biguanide class that is considered safe for use during pregnancy, represents another potential alternative for future management of LAM, either as monotherapy or in combination with sirolimus, although its efficacy has not yet been definitively established [87].

Inhalation therapy is recommended for patients with impaired FEV1, as bronchodilators help alleviate symptoms, particularly in those with reversible obstructive ventilatory dysfunction. However, studies indicate that bronchodilators and inhaled corticosteroids do not impact disease progression [88].

In pregnant women with LAM, clinicians should maintain a high index of suspicion for pneumothorax, a complication that is most commonly managed through the insertion of a pleural tube, which is the preferred intervention in 57% of cases. Other therapeutic alternatives include surgical pleurodesis (29%), chemical pleurodesis (5%), or close observation (10%) [35].

Surgical interventions described for cases of chylothorax during pregnancy involve left thoracotomy and the ligation of multiple lymphatic channels near the esophageal hiatus and in the area between the aorta and the azygos vein, leading to symptomatic and imaging improvement postoperatively [81]. However, these therapeutic procedures are reserved for carefully selected cases.

In general, management should be individualized based on the patient’s disease severity and overall health. It is important to tailor the treatment plan to the specific needs of the patient, taking into account the risks associated with LAM (Table 2). For patients with advanced or progressive LAM, it is essential to provide detailed counseling to ensure that the patient fully understands the significant health risks posed to both herself and the fetus. This information should be communicated clearly to facilitate informed decision-making. Care for these patients should be managed through a multidisciplinary approach, involving pulmonologists, obstetricians, and critical care specialists to address the complexity of the disease and its potential impact during pregnancy. In patients with TSC, genetic counseling should be offered before conception to assess potential hereditary risks and the implications for the fetus. This is a crucial step in preparing both the patient and her family for possible genetic considerations. For patients with mild LAM, it is important to educate them about the increased risks during pregnancy. These risks include the possibility of pneumothorax, chylous effusions, bleeding from angiomyolipomas, and disease progression. Patients should also undergo baseline clinical evaluations, including imaging studies and pulmonary function tests, to assess their health status before conception. Regarding medication, the use of mTOR inhibitors such as sirolimus during pregnancy is generally avoided due to a lack of sufficient data on their safety. If the patient’s clinical condition deteriorates significantly, however, further studies are needed to assess whether mTOR inhibitors could be used safely under careful monitoring. Throughout the pregnancy, regular monitoring is critical, and the care team should include both a pulmonologist and an obstetrician experienced in managing LAM. This ensures that the patient receives comprehensive care for both maternal and fetal health and that potential complications are addressed promptly.

## 14. Conclusions

In conclusion, the management of LAM during pregnancy necessitates a nuanced and individualized approach, owing to the progressive course of the disease and its potential complications. Comprehensive multidisciplinary care and meticulous family planning are critical to mitigating risks and enhancing the quality of life for affected patients.

In summary, while many women with LAM can carry a pregnancy to term without significant complications, the condition is generally considered a relative contraindication due to associated risks, including spontaneous abortion, medically induced terminations, and respiratory exacerbations. Research suggests that pregnancy outcomes may vary, with women diagnosed with LAM during pregnancy facing higher risks of adverse outcomes compared to those diagnosed before or after pregnancy. Additionally, severe complications such as atraumatic renal angiomyolipoma rupture can arise, underscoring the importance of a careful, individualized approach. Comprehensive multidisciplinary management and proactive family planning are essential to optimizing outcomes and improving the quality of life for patients with LAM during pregnancy.

## Figures and Tables

**Table 1:** Case reports from the literature, where LAM was diagnosed during pregnancy.

Nr.Crt.	Authors, Year	Maternal Age (Years)	Symptoms During Pregnancy	Diagnosis Preceding Pregnancy?	Abdominopelvic Involvement	Sirolimus Treatment Before Pregnancy	Sirolimus in Pregnancy	Complications of LAM During Pregnancy	Other Pregnancy Complications	Treatment and/or Interventions	Gestational Age at Delivery (Years)	Delivery Mode	Neonatal Outcome	Functional Deterioration During/After Pregnancy
1	Eileen Wang-Koehler et al., 2024 [75]	37	Right-sided chest pain	No	Renal angiomyolipomas	No	No	Recurrent right pneumothorax	Severe preeclampsia	Chest tube	30	Cesarean	Healthy	No
			Abdominal pain					Chylous ascites	Atrial fibrillation	Betamethasone				
			Constipation						Pulmonary embolism	Magnesium				
									Bowel dilation and perforation	Hydromorphone patient-controlled analgesia (PCA) pump				
										Intravenous labetalol				
2	Yashi Zhu et al., 2023 [76]	31	Asymptomatic	No	Retroperitoneal tumor	No	No	No	No	Complete curettage	13	Curettage		No
3	Lily Alkemade et al., 2021 [77]	36	Acute onset dyspnea	No	No	No	No	Small pneumothorax on the left apical side	No	Observation	39	Cesarean	Healthy	No
			Stabbing pain in the chest and left shoulder											
			Cough											
4	Soraya Saleh Gargari et al., 2009 [78]	35	Mild nonprogressive exertional dyspnea	No	No	No	No	No	No	No	37	Cesarean	Healthy	
5	M Faehling et al., 2011 [79]	29	Gradually increasing dyspnea		No	Yes	Discontinued (6 weeks + 0)	Left pneumothorax	No	Chest tube	32	Cesarean	Healthy	No
			Cough				Restarted (23 weeks + 0) at 2 mg/day			Betamethasone				
6	Johnston CR et al., 2010 [80]	18	Dyspnea	No	Renal angiomyolipoma	No	No	Bilateral pneumothorax	No	Chest tube	29	Cesarean	Poor in utero fetal growth	Yes
			Fatigue											
7	Brunelli A et al., 1996 [81]	26	Severe dyspnea	No	No	No	No	Bilateral chylothorax	No	Left thoracotomy, Ligation of multiple small lymphatic channels	35	Cesarean	Healthy	No
8	Cihan Çetin et al., 2015 [41]	26	Left flank pain	No	Renal angiomyolipomas	No	No	No	No	No	36	Vaginally	Healthy	No
9	Kazuhiro Toyoda et al., 2006 [82]	26	Dry cough	No	Renal angiomyolipomas	No	No	Left pneumothorax	No	Chest tube	32	Cesarean	Healthy	No
			Sudden onset of hemosputum											
			Left chest pain											
10	Ruth McCartney et al., 2009 [83]	30	Dyspnea	No	No	No	No	Recurrent bilateral pneumothorax	No	Chest tube	34	Cesarean	Healthy	No
			Left-sided pleuritic chest pain											

**Table 2:** Advising for Decision-Making Regarding Pregnancy in LAM.

Patients with advanced/progressive disease	Counseling to raise awareness of the significant health risks to both the patient and the fetus
Patients with TSC	Genetic counseling prior to conception
Patients with mild disease	Informing the patient about the increased risks of pneumothorax, chylous effusion, bleeding from angiomyolipoma, or disease progression
	Clinical, radiological, and respiratory function assessments before the start of the pregnancy
	The administration of mTOR inhibitors (sirolimus) during pregnancy requires further studies and is currently avoided unless the patient’s clinical condition deteriorates significantly
	Monitoring during pregnancy by a pulmonologist and an obstetrician specialized in LAM.

## Data Availability

Data are available by emailing the corresponding author.
